# Aqua­(propane­dioato-κ^2^ *O* ^1^,*O* ^3^)[2-(1*H*-pyrazol-1-yl-κ*N* ^2^)-1,10-phenanthroline-κ^2^ *N*,*N*′]nickel(II) trihydrate

**DOI:** 10.1107/S1600536809017188

**Published:** 2009-05-14

**Authors:** Huai Yi Yan, Tai Qiu Hu, Jing Min Shi

**Affiliations:** a Department of Chemistry, Xinzhou Teacher’s University, Shanxi Xinzhou 034000, People’s Republic of China; b Department of Chemistry, Shandong Normal University, Jinan 250014, People’s Republic of China

## Abstract

In the title mononuclear complex, [Ni(C_3_H_2_O_4_)(C_15_H_10_N_4_)(H_2_O)]·3H_2_O, the metal center is coordinated in a distorted NiN_3_O_3_ geometry. In the crystal structure, inter­molecular O—H⋯O hydrogen bonds link the components into a two-dimensional network. In addition, there are weak π–π stacking inter­actions between symmetry-related phenanthroline rings, with a centroid–centroid distance of 3.6253 (17) Å.

## Related literature

For a related Ni^II^ structure with 1,10-phenanthroline, see: Zhang *et al.* (2008 ▶).
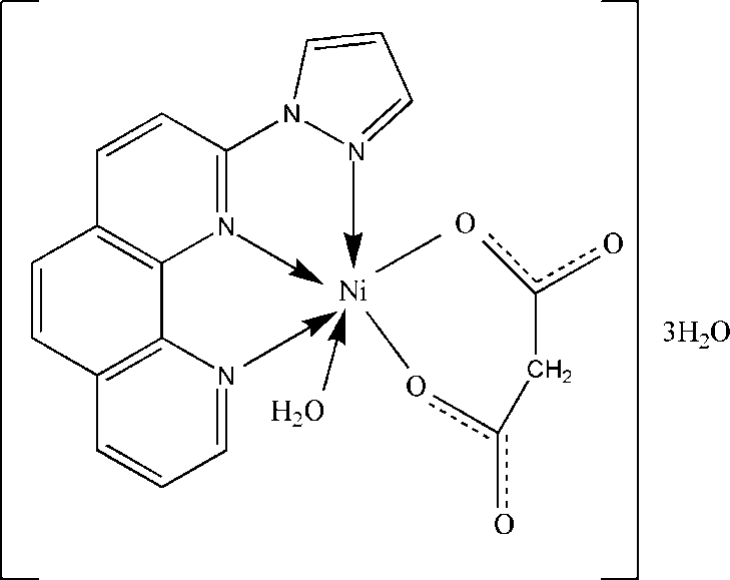


## Experimental

### 

#### Crystal data

[Ni(C_3_H_2_O_4_)(C_15_H_10_N_4_)(H_2_O)]·3H_2_O
*M* *_r_* = 479.09
Triclinic, 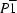

*a* = 7.8066 (14) Å
*b* = 11.639 (2) Å
*c* = 12.159 (2) Å
α = 109.234 (2)°
β = 103.493 (2)°
γ = 90.601 (2)°
*V* = 1009.8 (3) Å^3^
*Z* = 2
Mo *K*α radiation
μ = 1.02 mm^−1^
*T* = 298 K
0.31 × 0.21 × 0.15 mm

#### Data collection

Bruker SMART APEX CCD diffractometer
Absorption correction: multi-scan (*SADABS*; Sheldrick, 1996 ▶) *T*_min_ = 0.744, *T*_max_ = 0.863
5558 measured reflections
3887 independent reflections
3414 reflections with *I* > 2σ(*I*)
*R*_int_ = 0.018

#### Refinement

*R*[*F* ^2^ > 2σ(*F* ^2^)] = 0.040
*wR*(*F* ^2^) = 0.107
*S* = 1.06
3887 reflections
280 parameters
3 restraints
H-atom parameters constrained
Δρ_max_ = 0.41 e Å^−3^
Δρ_min_ = −0.36 e Å^−3^

### 

Data collection: *SMART* (Bruker, 1997 ▶); cell refinement: *SAINT* (Bruker, 1997 ▶); data reduction: *SAINT*; program(s) used to solve structure: *SHELXTL* (Sheldrick, 2008 ▶); program(s) used to refine structure: *SHELXTL*; molecular graphics: *SHELXTL*; software used to prepare material for publication: *SHELXTL*.

## Supplementary Material

Crystal structure: contains datablocks I, global. DOI: 10.1107/S1600536809017188/lh2813sup1.cif

Structure factors: contains datablocks I. DOI: 10.1107/S1600536809017188/lh2813Isup2.hkl

Additional supplementary materials:  crystallographic information; 3D view; checkCIF report

## Figures and Tables

**Table:** 

N1—Ni1	2.186 (2)
N3—Ni1	2.007 (2)
N4—Ni1	2.145 (2)
Ni1—O3	2.0149 (18)
Ni1—O1	2.0427 (17)
Ni1—O5	2.0603 (18)

**Table:** 

N3—Ni1—O3	172.30 (8)
N3—Ni1—O1	89.69 (7)
O3—Ni1—O1	89.36 (7)
N3—Ni1—O5	94.60 (8)
O3—Ni1—O5	86.90 (8)
O1—Ni1—O5	174.18 (7)
N3—Ni1—N4	78.21 (8)
O3—Ni1—N4	94.31 (8)
O1—Ni1—N4	96.83 (7)
O5—Ni1—N4	87.90 (8)
N3—Ni1—N1	75.03 (8)
O3—Ni1—N1	112.52 (8)
O1—Ni1—N1	85.91 (8)
O5—Ni1—N1	91.40 (8)
N4—Ni1—N1	153.09 (8)

**Table 2:** Hydrogen-bond geometry (Å, °)

*D*—H⋯*A*	*D*—H	H⋯*A*	*D*⋯*A*	*D*—H⋯*A*
O5—H2⋯O2^i^	0.87	1.77	2.622 (3)	170
O7—H15⋯O8^ii^	0.82	2.04	2.791 (4)	152
O5—H1⋯O6^iii^	0.82	1.97	2.773 (3)	167
O6—H12⋯O3^iv^	0.89	2.00	2.785 (3)	147
O8—H3⋯O4	0.90	2.13	2.895 (4)	143
O7—H8⋯O4	0.90	1.99	2.836 (4)	155
O6—H11⋯O8	0.76	1.99	2.736 (4)	165

Symmetry codes: (i) 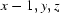; (ii) 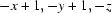; (iii) 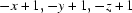; (iv) 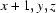.

**Fig. 1.**
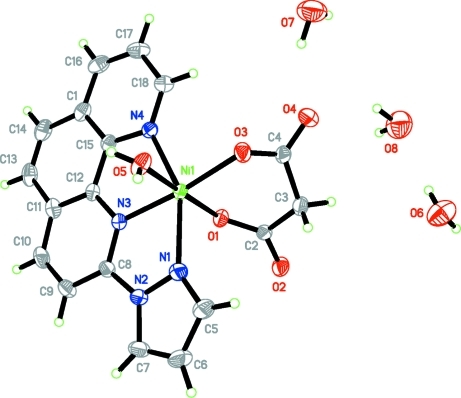
The molecular structure of (I) showing the atom numbering scheme with thermal ellipsoids drawn at the 30% probability level.

**Fig. 2.**
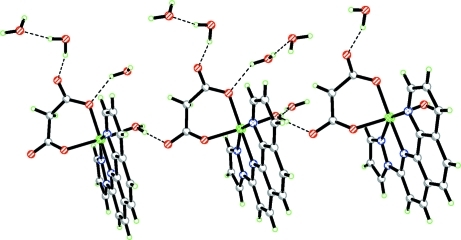
Part of the crystal structure with hydrogen bonds drawn as dashed lines.

## supplementary crystallographic information

### Comment

Metal complexes containing derivatives of 1,10-phenanthroline as ligands play a pivotal role in the area of modern coordination chemistry. The interest in this area has caused us to synthesize the title complex, and here we report its crystal structure, (I), Fig. 1.

The coordination bond lengths and associated angles (Table 1) indicate that the Ni^II^ ion assumes a distorted octahedral geometry. All non-hydrogen atoms of the ligand 2-(1*H*-pyrazol-1-yl)-1,10-phenanthroline define a plane within 0.0368 Å with a maximum deviation of -0.0854 (19) Å for atom N4. In the crystal structure, intermolecular O—H···O hydrogen bonds link the components of the structure into a two-dimensional network (see Fig. 2 and Table 2). In addition, there are weak π–π stacking interactions with *Cg*1···*Cg*2^i^ = 3.6253 (17) Å and *Cg*1···*Cg*2^i^_perp_ = 3.411 Å; α is 1.41° [symmetry code: (i) -*x*, -*y*, -*z*; *Cg*1 and *Cg*2 are the centroids of C1/C15–C18/N4 ring and C1/C11–C15 ring, respectively; *Cg*1···*Cg*2_perp_ is the perpendicular distance from ring *Cg*1 to ring *Cg*2^i^; α is the dihedral angle between the *Cg*1 ring plane and  the *Cg*2 ring plane].

### Experimental

A 5 ml H_2_O solution of hydrated nickel perchlorate (0.1606 g, 0.439 mmol) was added to an ethanol solution containing 2-(1*H*-pyrazol-1-yl)1,10-phenanthroline (0.1025 g, 0.416 mmol) and the solution was stirred for a few minutes. A 5 ml H_2_O solution of sodium propanedioate (0.0714 g, 0.482 mmol) was then added dropwise into the above solution. The solution was stirred for another a few minutes. Blue single crystals were obtained after the filtrate had been allowed to stand at room temperature for two weeks.

### Refinement

H atoms of water molecules were located in a difference Fourier map and refined as riding in their as-found positions, with *U*_iso_(H) = 1.5*U*_eq_(O). All other H atoms were placed in calculated positions, and refined as riding, with C—H = 0.97 Å for methylene and C—H = 0.93 Å for other H atoms, *U*_iso_(H) = 1.2_eq_(C).

### Crystal data

**Table:**

[Ni(C_3_H_2_O_4_)(C_15_H_10_N_4_)(H_2_O)]·3H_2_O	*Z* = 2
*M**_r_* = 479.09	*F*(000) = 496
Triclinic, *P*1	*D*_x_ = 1.576 Mg m^−^^3^
Hall symbol: -P 1	Mo *K*α radiation, λ = 0.71073 Å
*a* = 7.8066 (14) Å	Cell parameters from 2439 reflections
*b* = 11.639 (2) Å	θ = 2.7–26.4°
*c* = 12.159 (2) Å	µ = 1.02 mm^−^^1^
α = 109.234 (2)°	*T* = 298 K
β = 103.493 (2)°	Block, blue
γ = 90.601 (2)°	0.31 × 0.21 × 0.15 mm
*V* = 1009.8 (3) Å^3^	

### Data collection

**Table:**

Bruker SMART APEX CCD diffractometer	3887 independent reflections
Radiation source: fine-focus sealed tube	3414 reflections with *I* > 2σ(*I*)
graphite	*R*_int_ = 0.018
φ and ω scans	θ_max_ = 26.0°, θ_min_ = 1.8°
Absorption correction: multi-scan (*SADABS*; Sheldrick, 1996)	*h* = −9→9
*T*_min_ = 0.744, *T*_max_ = 0.863	*k* = −10→14
5558 measured reflections	*l* = −14→12

### Refinement

**Table:**

Refinement on *F*^2^	Primary atom site location: structure-invariant direct methods
Least-squares matrix: full	Secondary atom site location: difference Fourier map
*R*[*F*^2^ > 2σ(*F*^2^)] = 0.040	Hydrogen site location: inferred from neighbouring sites
*wR*(*F*^2^) = 0.107	H-atom parameters constrained
*S* = 1.06	*w* = 1/[σ^2^(*F*_o_^2^) + (0.0609*P*)^2^ + 0.1787*P*] where *P* = (*F*_o_^2^ + 2*F*_c_^2^)/3
3887 reflections	(Δ/σ)_max_ < 0.001
280 parameters	Δρ_max_ = 0.41 e Å^−^^3^
3 restraints	Δρ_min_ = −0.36 e Å^−^^3^

### Special details

**Table:**

Geometry. All e.s.d.'s (except the e.s.d. in the dihedral angle between two l.s. planes) are estimated using the full covariance matrix. The cell e.s.d.'s are taken into account individually in the estimation of e.s.d.'s in distances, angles and torsion angles; correlations between e.s.d.'s in cell parameters are only used when they are defined by crystal symmetry. An approximate (isotropic) treatment of cell e.s.d.'s is used for estimating e.s.d.'s involving l.s. planes.
Refinement. Refinement of *F*^2^ against ALL reflections. The weighted *R*-factor *wR* and goodness of fit *S* are based on *F*^2^, conventional *R*-factors *R* are based on *F*, with *F* set to zero for negative *F*^2^. The threshold expression of *F*^2^ > σ(*F*^2^) is used only for calculating *R*-factors(gt) *etc*. and is not relevant to the choice of reflections for refinement. *R*-factors based on *F*^2^ are statistically about twice as large as those based on *F*, and *R*- factors based on ALL data will be even larger.

### Fractional atomic coordinates and isotropic or equivalent isotropic displacement parameters (Å^2^)

**Table:**

	*x*	*y*	*z*	*U*_iso_*/*U*_eq_	
C1	−0.2105 (3)	−0.0069 (3)	0.0485 (2)	0.0402 (6)	
C2	0.5533 (3)	0.2194 (2)	0.3192 (2)	0.0317 (5)	
C3	0.5634 (4)	0.3565 (2)	0.3449 (3)	0.0481 (7)	
H3A	0.6004	0.3975	0.4313	0.058*	
H3B	0.6542	0.3783	0.3108	0.058*	
C4	0.3933 (3)	0.4043 (2)	0.2967 (3)	0.0391 (6)	
C5	0.4542 (4)	0.2600 (3)	0.6200 (2)	0.0501 (7)	
H5	0.5037	0.3403	0.6456	0.060*	
C6	0.4961 (4)	0.1811 (3)	0.6853 (3)	0.0585 (8)	
H6	0.5771	0.1983	0.7594	0.070*	
C7	0.3961 (4)	0.0755 (3)	0.6196 (3)	0.0485 (7)	
H7	0.3934	0.0053	0.6399	0.058*	
C8	0.1738 (3)	0.0109 (2)	0.4191 (2)	0.0349 (5)	
C9	0.1191 (4)	−0.1082 (3)	0.4083 (3)	0.0464 (7)	
H9	0.1665	−0.1405	0.4681	0.056*	
C10	−0.0063 (4)	−0.1753 (3)	0.3072 (3)	0.0500 (7)	
H10	−0.0437	−0.2549	0.2977	0.060*	
C11	−0.0800 (4)	−0.1258 (2)	0.2169 (3)	0.0423 (6)	
C12	−0.0145 (3)	−0.0073 (2)	0.2364 (2)	0.0334 (5)	
C13	−0.2146 (4)	−0.1856 (3)	0.1088 (3)	0.0513 (8)	
H13	−0.2603	−0.2650	0.0932	0.062*	
C14	−0.2756 (4)	−0.1290 (3)	0.0300 (3)	0.0501 (7)	
H14	−0.3633	−0.1704	−0.0391	0.060*	
C15	−0.0762 (3)	0.0535 (2)	0.1524 (2)	0.0321 (5)	
C16	−0.2689 (4)	0.0593 (3)	−0.0293 (2)	0.0490 (7)	
H16	−0.3580	0.0242	−0.0994	0.059*	
C17	−0.1951 (4)	0.1745 (3)	−0.0018 (2)	0.0506 (7)	
H17	−0.2345	0.2190	−0.0523	0.061*	
C18	−0.0589 (4)	0.2261 (3)	0.1032 (2)	0.0411 (6)	
H18	−0.0085	0.3047	0.1202	0.049*	
N1	0.3355 (3)	0.2062 (2)	0.51739 (19)	0.0395 (5)	
N2	0.2992 (3)	0.0911 (2)	0.51724 (19)	0.0366 (5)	
N3	0.1093 (3)	0.05894 (19)	0.33639 (18)	0.0317 (4)	
N4	0.0004 (3)	0.16792 (19)	0.17875 (18)	0.0325 (5)	
Ni1	0.19360 (4)	0.22721 (3)	0.34854 (3)	0.02967 (12)	
O1	0.4043 (2)	0.15843 (15)	0.28425 (15)	0.0354 (4)	
O2	0.6966 (2)	0.17407 (18)	0.3347 (2)	0.0496 (5)	
O3	0.2619 (2)	0.38952 (16)	0.33705 (18)	0.0437 (5)	
O4	0.3907 (3)	0.4573 (2)	0.2239 (2)	0.0593 (6)	
O5	−0.0012 (2)	0.31123 (18)	0.42578 (17)	0.0474 (5)	
H2	−0.0974	0.2633	0.3875	0.071*	
H1	0.0036	0.3463	0.4970	0.071*	
O6	0.9811 (4)	0.5352 (2)	0.3413 (2)	0.0834 (8)	
H12	1.0838	0.5098	0.3269	0.125*	
H11	0.9107	0.5181	0.2821	0.125*	
O7	0.1756 (4)	0.5519 (3)	0.0576 (3)	0.0948 (9)	
H8	0.2184	0.5035	0.1004	0.142*	
H15	0.2443	0.5350	0.0145	0.142*	
O8	0.6999 (4)	0.5114 (3)	0.1500 (3)	0.0967 (10)	
H4	0.6894	0.4311	0.1063	0.145*	
H3	0.5869	0.5176	0.1541	0.145*	

### Atomic displacement parameters (Å^2^)

**Table:**

	*U*^11^	*U*^22^	*U*^33^	*U*^12^	*U*^13^	*U*^23^
C1	0.0324 (14)	0.0451 (15)	0.0345 (13)	0.0010 (11)	0.0083 (11)	0.0025 (12)
C2	0.0300 (13)	0.0359 (13)	0.0310 (12)	0.0031 (10)	0.0078 (10)	0.0139 (10)
C3	0.0311 (14)	0.0379 (15)	0.075 (2)	−0.0006 (11)	0.0046 (13)	0.0257 (15)
C4	0.0322 (14)	0.0281 (13)	0.0527 (16)	−0.0026 (10)	0.0022 (12)	0.0142 (12)
C5	0.0474 (17)	0.0535 (18)	0.0400 (15)	−0.0024 (14)	−0.0011 (13)	0.0122 (14)
C6	0.0524 (19)	0.078 (2)	0.0394 (16)	0.0021 (17)	−0.0043 (14)	0.0244 (16)
C7	0.0488 (17)	0.0619 (19)	0.0444 (15)	0.0125 (15)	0.0096 (13)	0.0319 (15)
C8	0.0347 (14)	0.0368 (13)	0.0377 (13)	0.0047 (11)	0.0115 (11)	0.0169 (11)
C9	0.0527 (18)	0.0428 (16)	0.0541 (17)	0.0055 (13)	0.0154 (14)	0.0289 (14)
C10	0.0561 (19)	0.0337 (15)	0.0650 (19)	−0.0046 (13)	0.0204 (15)	0.0197 (14)
C11	0.0445 (16)	0.0366 (14)	0.0471 (15)	−0.0010 (12)	0.0181 (13)	0.0116 (12)
C12	0.0281 (13)	0.0340 (13)	0.0376 (13)	−0.0006 (10)	0.0106 (10)	0.0100 (11)
C13	0.0506 (18)	0.0386 (15)	0.0545 (18)	−0.0165 (13)	0.0142 (14)	0.0026 (14)
C14	0.0416 (17)	0.0502 (17)	0.0418 (15)	−0.0104 (13)	0.0044 (13)	−0.0015 (13)
C15	0.0296 (13)	0.0354 (13)	0.0305 (12)	0.0027 (10)	0.0102 (10)	0.0083 (10)
C16	0.0425 (16)	0.0588 (19)	0.0332 (14)	0.0082 (14)	0.0000 (12)	0.0057 (13)
C17	0.0570 (19)	0.0522 (18)	0.0369 (15)	0.0138 (15)	0.0006 (13)	0.0154 (13)
C18	0.0436 (15)	0.0408 (15)	0.0385 (14)	0.0085 (12)	0.0076 (12)	0.0148 (12)
N1	0.0396 (13)	0.0398 (12)	0.0373 (12)	0.0003 (10)	0.0046 (10)	0.0145 (10)
N2	0.0352 (12)	0.0426 (12)	0.0371 (11)	0.0056 (10)	0.0079 (9)	0.0208 (10)
N3	0.0294 (11)	0.0324 (11)	0.0347 (11)	0.0007 (8)	0.0081 (8)	0.0133 (9)
N4	0.0302 (11)	0.0343 (11)	0.0326 (11)	0.0033 (9)	0.0070 (8)	0.0114 (9)
Ni1	0.02559 (19)	0.02943 (19)	0.03366 (19)	0.00035 (12)	0.00542 (13)	0.01179 (14)
O1	0.0286 (9)	0.0319 (9)	0.0421 (10)	0.0002 (7)	0.0076 (7)	0.0090 (8)
O2	0.0311 (10)	0.0451 (11)	0.0792 (14)	0.0093 (8)	0.0159 (10)	0.0282 (10)
O3	0.0369 (11)	0.0334 (10)	0.0658 (13)	0.0051 (8)	0.0160 (9)	0.0210 (9)
O4	0.0512 (13)	0.0661 (14)	0.0783 (15)	0.0069 (11)	0.0143 (11)	0.0488 (13)
O5	0.0315 (10)	0.0577 (12)	0.0436 (10)	0.0017 (9)	0.0104 (8)	0.0043 (9)
O6	0.0684 (17)	0.0799 (19)	0.0820 (17)	0.0267 (14)	0.0127 (14)	0.0053 (15)
O7	0.090 (2)	0.123 (3)	0.097 (2)	0.0304 (19)	0.0232 (17)	0.070 (2)
O8	0.074 (2)	0.128 (3)	0.105 (2)	0.0108 (18)	0.0367 (17)	0.051 (2)

### Geometric parameters (Å, °)

**Table:**

C1—C15	1.404 (4)	C12—N3	1.351 (3)
C1—C16	1.408 (4)	C12—C15	1.425 (4)
C1—C14	1.432 (4)	C13—C14	1.336 (5)
C2—O2	1.243 (3)	C13—H13	0.9300
C2—O1	1.261 (3)	C14—H14	0.9300
C2—C3	1.519 (4)	C15—N4	1.358 (3)
C3—C4	1.511 (4)	C16—C17	1.356 (4)
C3—H3A	0.9700	C16—H16	0.9300
C3—H3B	0.9700	C17—C18	1.403 (4)
C4—O4	1.231 (3)	C17—H17	0.9300
C4—O3	1.269 (3)	C18—N4	1.317 (3)
C5—N1	1.320 (4)	C18—H18	0.9300
C5—C6	1.395 (4)	N1—N2	1.367 (3)
C5—H5	0.9300	N1—Ni1	2.186 (2)
C6—C7	1.341 (5)	N3—Ni1	2.007 (2)
C6—H6	0.9300	N4—Ni1	2.145 (2)
C7—N2	1.366 (3)	Ni1—O3	2.0149 (18)
C7—H7	0.9300	Ni1—O1	2.0427 (17)
C8—N3	1.311 (3)	Ni1—O5	2.0603 (18)
C8—N2	1.398 (3)	O5—H2	0.8653
C8—C9	1.401 (4)	O5—H1	0.8184
C9—C10	1.367 (4)	O6—H12	0.8934
C9—H9	0.9300	O6—H11	0.7604
C10—C11	1.414 (4)	O7—H8	0.9033
C10—H10	0.9300	O7—H15	0.8159
C11—C12	1.390 (4)	O8—H4	0.9009
C11—C13	1.433 (4)	O8—H3	0.8976
			
C15—C1—C16	116.2 (3)	N4—C15—C12	117.1 (2)
C15—C1—C14	118.1 (3)	C1—C15—C12	119.0 (2)
C16—C1—C14	125.7 (3)	C17—C16—C1	119.9 (3)
O2—C2—O1	123.8 (2)	C17—C16—H16	120.1
O2—C2—C3	116.5 (2)	C1—C16—H16	120.1
O1—C2—C3	119.7 (2)	C16—C17—C18	119.6 (3)
C4—C3—C2	115.2 (2)	C16—C17—H17	120.2
C4—C3—H3A	108.5	C18—C17—H17	120.2
C2—C3—H3A	108.5	N4—C18—C17	122.8 (3)
C4—C3—H3B	108.5	N4—C18—H18	118.6
C2—C3—H3B	108.5	C17—C18—H18	118.6
H3A—C3—H3B	107.5	C5—N1—N2	104.6 (2)
O4—C4—O3	124.0 (3)	C5—N1—Ni1	144.3 (2)
O4—C4—C3	119.0 (3)	N2—N1—Ni1	110.94 (15)
O3—C4—C3	117.0 (2)	C7—N2—N1	111.0 (2)
N1—C5—C6	111.4 (3)	C7—N2—C8	130.7 (2)
N1—C5—H5	124.3	N1—N2—C8	118.3 (2)
C6—C5—H5	124.3	C8—N3—C12	119.8 (2)
C7—C6—C5	106.2 (3)	C8—N3—Ni1	122.53 (18)
C7—C6—H6	126.9	C12—N3—Ni1	117.64 (16)
C5—C6—H6	126.9	C18—N4—C15	117.5 (2)
C6—C7—N2	106.8 (3)	C18—N4—Ni1	130.64 (19)
C6—C7—H7	126.6	C15—N4—Ni1	111.70 (16)
N2—C7—H7	126.6	N3—Ni1—O3	172.30 (8)
N3—C8—N2	112.9 (2)	N3—Ni1—O1	89.69 (7)
N3—C8—C9	122.4 (2)	O3—Ni1—O1	89.36 (7)
N2—C8—C9	124.7 (2)	N3—Ni1—O5	94.60 (8)
C10—C9—C8	118.0 (3)	O3—Ni1—O5	86.90 (8)
C10—C9—H9	121.0	O1—Ni1—O5	174.18 (7)
C8—C9—H9	121.0	N3—Ni1—N4	78.21 (8)
C9—C10—C11	121.1 (3)	O3—Ni1—N4	94.31 (8)
C9—C10—H10	119.5	O1—Ni1—N4	96.83 (7)
C11—C10—H10	119.5	O5—Ni1—N4	87.90 (8)
C12—C11—C10	116.0 (3)	N3—Ni1—N1	75.03 (8)
C12—C11—C13	117.6 (3)	O3—Ni1—N1	112.52 (8)
C10—C11—C13	126.4 (3)	O1—Ni1—N1	85.91 (8)
N3—C12—C11	122.7 (2)	O5—Ni1—N1	91.40 (8)
N3—C12—C15	115.3 (2)	N4—Ni1—N1	153.09 (8)
C11—C12—C15	122.0 (2)	C2—O1—Ni1	121.73 (15)
C14—C13—C11	121.1 (3)	C4—O3—Ni1	121.25 (16)
C14—C13—H13	119.4	Ni1—O5—H2	105.6
C11—C13—H13	119.4	Ni1—O5—H1	128.8
C13—C14—C1	122.2 (3)	H2—O5—H1	113.1
C13—C14—H14	118.9	H12—O6—H11	109.3
C1—C14—H14	118.9	H8—O7—H15	95.2
N4—C15—C1	124.0 (2)	H4—O8—H3	97.9
			
O2—C2—C3—C4	165.4 (2)	C15—C12—N3—C8	179.8 (2)
O1—C2—C3—C4	−14.8 (4)	C11—C12—N3—Ni1	178.57 (19)
C2—C3—C4—O4	−120.0 (3)	C15—C12—N3—Ni1	−2.4 (3)
C2—C3—C4—O3	61.9 (3)	C17—C18—N4—C15	−0.4 (4)
N1—C5—C6—C7	−0.8 (4)	C17—C18—N4—Ni1	−176.1 (2)
C5—C6—C7—N2	0.8 (4)	C1—C15—N4—C18	1.7 (3)
N3—C8—C9—C10	0.0 (4)	C12—C15—N4—C18	−178.3 (2)
N2—C8—C9—C10	179.4 (3)	C1—C15—N4—Ni1	178.24 (19)
C8—C9—C10—C11	−0.7 (4)	C12—C15—N4—Ni1	−1.8 (3)
C9—C10—C11—C12	1.4 (4)	C8—N3—Ni1—O1	81.8 (2)
C9—C10—C11—C13	−178.4 (3)	C12—N3—Ni1—O1	−95.92 (18)
C10—C11—C12—N3	−1.4 (4)	C8—N3—Ni1—O5	−94.3 (2)
C13—C11—C12—N3	178.3 (2)	C12—N3—Ni1—O5	88.01 (18)
C10—C11—C12—C15	179.6 (2)	C8—N3—Ni1—N4	178.8 (2)
C13—C11—C12—C15	−0.6 (4)	C12—N3—Ni1—N4	1.12 (17)
C12—C11—C13—C14	−0.4 (4)	C8—N3—Ni1—N1	−4.07 (19)
C10—C11—C13—C14	179.4 (3)	C12—N3—Ni1—N1	178.23 (19)
C11—C13—C14—C1	0.2 (5)	C18—N4—Ni1—N3	176.3 (2)
C15—C1—C14—C13	1.0 (4)	C15—N4—Ni1—N3	0.39 (15)
C16—C1—C14—C13	−179.3 (3)	C18—N4—Ni1—O3	−5.6 (2)
C16—C1—C15—N4	−1.7 (4)	C15—N4—Ni1—O3	178.52 (15)
C14—C1—C15—N4	178.1 (2)	C18—N4—Ni1—O1	−95.4 (2)
C16—C1—C15—C12	178.3 (2)	C15—N4—Ni1—O1	88.65 (16)
C14—C1—C15—C12	−1.9 (4)	C18—N4—Ni1—O5	81.2 (2)
N3—C12—C15—N4	2.8 (3)	C15—N4—Ni1—O5	−94.75 (16)
C11—C12—C15—N4	−178.2 (2)	C18—N4—Ni1—N1	170.1 (2)
N3—C12—C15—C1	−177.2 (2)	C15—N4—Ni1—N1	−5.8 (3)
C11—C12—C15—C1	1.8 (4)	C5—N1—Ni1—N3	178.2 (4)
C15—C1—C16—C17	0.3 (4)	N2—N1—Ni1—N3	4.34 (15)
C14—C1—C16—C17	−179.5 (3)	C5—N1—Ni1—O3	−0.2 (4)
C1—C16—C17—C18	1.0 (4)	N2—N1—Ni1—O3	−174.04 (14)
C16—C17—C18—N4	−1.0 (5)	C5—N1—Ni1—O1	87.4 (3)
C6—C5—N1—N2	0.5 (3)	N2—N1—Ni1—O1	−86.44 (16)
C6—C5—N1—Ni1	−173.6 (2)	C5—N1—Ni1—O5	−87.4 (3)
C6—C7—N2—N1	−0.5 (3)	N2—N1—Ni1—O5	98.73 (16)
C6—C7—N2—C8	−179.5 (3)	C5—N1—Ni1—N4	−175.5 (3)
C5—N1—N2—C7	−0.1 (3)	N2—N1—Ni1—N4	10.6 (3)
Ni1—N1—N2—C7	176.26 (17)	O2—C2—O1—Ni1	143.1 (2)
C5—N1—N2—C8	179.1 (2)	C3—C2—O1—Ni1	−36.7 (3)
Ni1—N1—N2—C8	−4.6 (3)	N3—Ni1—O1—C2	−145.91 (18)
N3—C8—N2—C7	−179.5 (2)	O3—Ni1—O1—C2	41.73 (19)
C9—C8—N2—C7	1.1 (4)	N4—Ni1—O1—C2	136.00 (18)
N3—C8—N2—N1	1.6 (3)	N1—Ni1—O1—C2	−70.90 (18)
C9—C8—N2—N1	−177.9 (2)	O4—C4—O3—Ni1	135.1 (2)
N2—C8—N3—C12	−179.5 (2)	C3—C4—O3—Ni1	−46.9 (3)
C9—C8—N3—C12	0.0 (4)	O1—Ni1—O3—C4	1.6 (2)
N2—C8—N3—Ni1	2.8 (3)	O5—Ni1—O3—C4	177.2 (2)
C9—C8—N3—Ni1	−177.69 (19)	N4—Ni1—O3—C4	−95.2 (2)
C11—C12—N3—C8	0.8 (4)	N1—Ni1—O3—C4	86.9 (2)

### Hydrogen-bond geometry (Å, °)

**Table:**

*D*—H···*A*	*D*—H	H···*A*	*D*···*A*	*D*—H···*A*
O5—H2···O2^i^	0.87	1.77	2.622 (3)	170
O7—H15···O8^ii^	0.82	2.04	2.791 (4)	152
O5—H1···O6^iii^	0.82	1.97	2.773 (3)	167
O6—H12···O3^iv^	0.89	2.00	2.785 (3)	147
O8—H3···O4	0.90	2.13	2.895 (4)	143
O7—H8···O4	0.90	1.99	2.836 (4)	155
O6—H11···O8	0.76	1.99	2.736 (4)	165

Symmetry codes: (i) *x*−1, *y*, *z*; (ii) −*x*+1, −*y*+1, −*z*; (iii) −*x*+1, −*y*+1, −*z*+1; (iv) *x*+1, *y*, *z*.
